# A hypoxia risk score for prognosis prediction and tumor microenvironment in adrenocortical carcinoma

**DOI:** 10.3389/fgene.2022.796681

**Published:** 2022-12-13

**Authors:** Yuanyuan Deng, Huihuang Li, Jinglan Fu, Ying Pu, Ying Zhang, Shijing Chen, Shiyu Tong, Huixia Liu

**Affiliations:** ^1^ Department of Geriatric Endocrine, Xiangya Hospital, Central South University, Changsha, China; ^2^ Department of Urology, Xiangya Hospital, Central South University, Changsha, China

**Keywords:** Adrenocortical carcinoma, hypoxia, risk score, tumor microenvironment, immunotherapy

## Abstract

**Background:** Adrenocortical carcinoma (ACC) is a rare malignant endocrine tumor derived from the adrenal cortex. Because of its highly aggressive nature, the prognosis of patients with adrenocortical carcinoma is not impressive. Hypoxia exists in the vast majority of solid tumors and contributes to invasion, metastasis, and drug resistance. This study aimed to reveal the role of hypoxia in Adrenocortical carcinoma and develop a hypoxia risk score (HRS) for Adrenocortical carcinoma prognostic prediction.

**Methods:** Hypoxia-related genes were obtained from the Molecular Signatures Database. The training cohorts of patients with adrenocortical carcinoma were downloaded from The Cancer Genome Atlas, while another three validation cohorts with comprehensive survival data were collected from the Gene Expression Omnibus. In addition, we constructed a hypoxia classifier using a random survival forest model. Moreover, we explored the relationship between the hypoxia risk score and immunophenotype in adrenocortical carcinoma to evaluate the efficacy of immune check inhibitors (ICI) therapy and prognosis of patients.

**Results:** HRS and tumor stage were identified as independent prognostic factors. HRS was negatively correlated with immune cycle activity, immune cell infiltration, and the T cell inflammatory score. Therefore, we considered the low hypoxia risk score group as the inflammatory immunophenotype, whereas the high HRS group was a non-inflammatory immunophenotype. In addition, the HRS was negatively related to the expression of common immune checkpoint molecules such as PD-L1, CD200, CTLA-4, and TIGIT, suggesting that patients with a lower hypoxia risk score respond better to immunotherapy.

**Conclusion:** We developed and validated a novel hypoxia risk score to predict the immunophenotype and response of patients with adrenocortical carcinoma to immune check inhibitors therapy. These findings not only provide fresh prognostic indicators for adrenocortical carcinoma but also offer several promising treatment targets for this disease.

## 1 Introduction

Adrenocortical carcinoma (ACC) is a rare endocrine malignant tumor that is accompanied by clinical manifestations caused by the excessive production of adrenal steroid hormones (Pittaway, 2019). The annual incidence of ACC is 1 -2 per million people, accounting for 0.2% of cancer deaths (Cronin et al., 2018). Moreover, the incidence peak in children younger than 10 years old and female aged 40–50 (Mcateer et al., 2013). Due to the highly invasive nature of ACC, the prognosis of patients is not optimistic even after radical surgery, which is the predominant treatment of ACC (Puglisi et al., 2020). Once metastasis and recurrence occur, the effects of endocrine therapy and chemotherapy are limited (Puglisi et al., 2018). Mitotane is the only drug widely accepted to be appliable for the treatment of patients with advanced or postoperative residual ACC, however, most patients experienced only temporary and partial remission (Puglisi et al., 2020). Identically, according to a multicenter study, the overall survival rate of mitotane in combination with EDP (etoposide, doxorubicin and doxorubicin) or streptomycin remains poor (Fassnacht et al., 2012). Hence, a more effective treatment is required for patients with ACC.

In recent years, immunotherapy has shown significant superiority in a variety of cancers (Guirgis, 2018; Nakamura, 2019; Marinelli et al., 2020), and the research of immunological events of ACC has progressed. The majority studies focused on the treatment outcome of programmed death-1 (PD-1) and programmed death-ligand 1 (PD-L1) expression, microsatellite instability (MSI) and tumor mutational burden (TMB) (Li et al., 2020). In the clinical trial of avelumab, the patients with higher PD-L1 mRNA expression were associated with stronger immune response (Billon et al., 2019). However, under the treatment of pembrolizumab, the expression of PD-1 was not apparently associated with the infiltrating immune cell of ACC patients (Habra et al., 2019). In addition to immune statues, the prognosis of various immunotherapies in ACC remains controversial (Fay et al., 2015; Raj et al., 2020). At this point, it is necessary to distinguish patients who are sensitive to immunotherapy. Furthermore, immune cells within the tumor microenvironment (TME) are indispensable in the process of recognizing and attacking cancer cells, which results in a powerful response to immunotherapy (Yost et al., 2019). Hypoxia is the most common condition in the TME because of rapid tumor cell proliferation and inadequate angiogenesis (Jing et al., 2019). Hypoxia contributes to the genetic and epigenetic changes that are associated with cellular biological behaviors and physiological functions, ultimately accelerating tumor generation, metastasis, and drug resistance. Increased hypoxia-inducible factor 1α (HIF-1α) expression, a critical hallmark of hypoxia, has been reported in various neoplasms, including lung carcinoma (Yang et al., 2016), breast cancer (De Heer et al., 2020) and melanoma (Malekan and Ebrahimzadeh, 2021). There is also increasing evidence indicating that HIF-α is a major regulator of immune actions (Jing et al., 2019). Non-etheless, the effect of hypoxia on ACC immunotherapy is unclear.

In this study, our purpose was to explore the role of hypoxia in the TME of ACC and its predictive effect. After unsupervised classification, the TCGA-ACC cohort was divided into two clusters. According to the immune statues in TME, the cluster 2 was considered as an inflammatory immune phenotype, whereas cluster 1 was a non-inflammatory immune phenotype. Then we conducted a hypoxia risk assessment and associated it with immunological and clinical characteristics. Subsequently, the correlation was analyzed between hypoxia risk score (HRS) and immune checkpoint inhibitor (ICI) response for providing an alternative treatment for ACC.

## 2 Methods and materials

### 2.1 Data collection and preprocessing

We downloaded the fragments per kilobase per million mapped fragments (FRKM) and clinical information of ACC patients from The Cancer Genome Atlas (TCGA) (https://portal.gdc.cancer.gov/). The FRKM was then rendered into a transcripts per kilobase million (TPM) value. Three validation cohorts with mRNA expression matrix and GPL data, including GSE19750 (GPL570), GSE33371 (GPL570), and GSE76019 (GPL13158), were retrieved from the Gene Expression Omnibus (GEO) (https://www.ncbi.nlm.nih.gov/geo/) by the “GEOquery” R package. Clinical data are summarized in Supplementary Table S1.

### 2.2 Unsupervised clustering for hypoxia-related genes

Hypoxia-related genes were collected from the Molecular Signatures Database (MSigDB, version 7.0). We included 191 hypoxia-related genes for further analysis because of nine gene symbol mismatches in TCGA-ACC (Supplementary Table S9). Unsupervised cluster was used to distinguish different hypoxia conditions by the Consensus Clustering algorithm (maxK = 5, reps = 100, pItem = 0.8, distance = “euclidean”, clusterAlg = “km”) implanted in the “ConsensusClusterPlus” R package (Kiselev and Andrews, 2019; Chengquan et al., 2021). Finally, TCGA-ACC cohort was divided into cluster 1 and cluster 2.

### 2.3 Identification of hypoxia-related differentially expressed genes (DEGs) and functional analysis

We applied the “LIMMA” package to determine DEGs between clusters 1 and 2. The filtering criteria of DEGs were false discovery rate adjusted *p* < 0.05 and absolute log2 fold change (log2 FC) > 1. Based on the identified genes, we then used Kyoto Encyclopedia of Genes and Genomes (KEGG) and Gene Ontology (GO) through the gene set enrichment analysis (GSEA), and the enrichment of the interferon-γ (IFN-γ) pathway between the two clusters was determined also using GSEA. The gene sets of KEGG and GO were downloaded from https://www.gsea-msigdb.org/gsea/index.jsp, and the IFN-γ gene sets were collected from Mariathasan’s study (Hänzelmann et al., 2013) (Supplementary Table S2).

### 2.4 Identification of immunological characteristics of the TME in ACC

There are several critical procedures included in the cancer immunity cycle, and the activities of these processes were gathered from http://biocc.hrbmu.edu.cn/TIP/. It can be roughly summarized as the release and presentation of cancer cell antigens (steps 1 and 2), activation of the immune response (step 3), recruitment and invasion of immune cells (steps 4 and 5), and identification and killing of cancer cells by effector T cells (steps 6 and 7) (Chen, 2013). We then estimated the abundance of tumor-infiltrating immune cells in the TME using a single-sample gene set enrichment analysis algorithm (ssGSEA) (Wang et al., 2020), and the relevant immune cell gene sets acquired from Charoentong et al. (2017) (Supplementary Table S3). For the sake of persuasion, we applied six other algorithms incorporated in TIMER (Li et al., 2017), EPIC (Yang et al., 2021a), Cibersort-ABS (Tan and Wang, 2022), Quan-Tiseq (Plattner et al., 2020), x-Cell (Tan and Wang, 2022), and Mcp-counter (Becht et al., 2016) to validate the tumor immune cell infiltration level of which EPIS, Quan-Tiseq, TIMER, and x-Cell were applied by the “immunedeconv” R package, while we downloaded LM22 from http://cibersort.stanford.edu/and used the “MCPcounter” R package for verification (Li et al., 2017; Plattner et al., 2020; Xu et al., 2021) (Supplementary Table S3). We also made the correlation analysis between MSI, TMB and HRS.

### 2.5 Generation and validation of the HRS

According to the univariate Cox analysis, we selected 143 DEGs as prognostic for hypoxia. Then, using the least absolute shrinkage and selector operation (LASSO) regression analysis and cross validation, we screened out six genes (SLC7A4, ISL1, SLC30A10, HOXC11, LHX2, and ZIC2) to calculate the HRS. The random survival forest model is an ensemble tree method used for survival analysis (Jamet et al., 2021). We generated the HRS on the basis of six genes with optimal predictive value using the rfsrc function performed by the “randomForestSRC” R package (kogalur.github.io/randomForestSRC) (Jamet et al., 2021). Taking the intermediate value of HRS as the cut-off point, we divided the cohort into high-risk and low-risk score groups. The Kaplan-Meier method and log rank test were applied for survival and statistical significance analyses between risk groups, respectively. Moreover, we evaluated the accuracy of the HRS using receiver operating characteristic curves visualized by the area under the curve. The prognostic value of the HRS was further verified using three external validation cohorts (GSE19750, GSE33371, and GSE76019).

### 2.6 Clinical characterizes combined with HRS for prognosis prediction

Multivariate Cox analyses were conducted to determine whether age, sex, tumor node metastasis stage, and HRS were prognostic factors for ACC. The results were displayed by the “forestplot” R package. The nomogram depicted the degree of contribution of the clinical factors and HRS to the survival probability at different time points. To further validate the predictive performance of nomogram, we made decision curve analysis (DCA).

### 2.7 Statistical analyses

We explored the correlation between immune status and HRS, and the expression of immune check point molecules and the HRS, using Spearman coefficients and a Pearson correlation analysis. Continuous variables between binary groups were compared using either the *t*-test or Mann-Whitney U test. Comparisons of classification variables were performed using Chi-square or Fisher tests. Statistical tests were two-sided, and the level of significance was set at *p* < 0.05. All statistical data analyses were performed using R software, version 4.0.3 (http:www.r-project.org).

## 3 Results

### 3.1 Hypoxia clusters correlated with immune phenotypes

**FIGURE 1:**
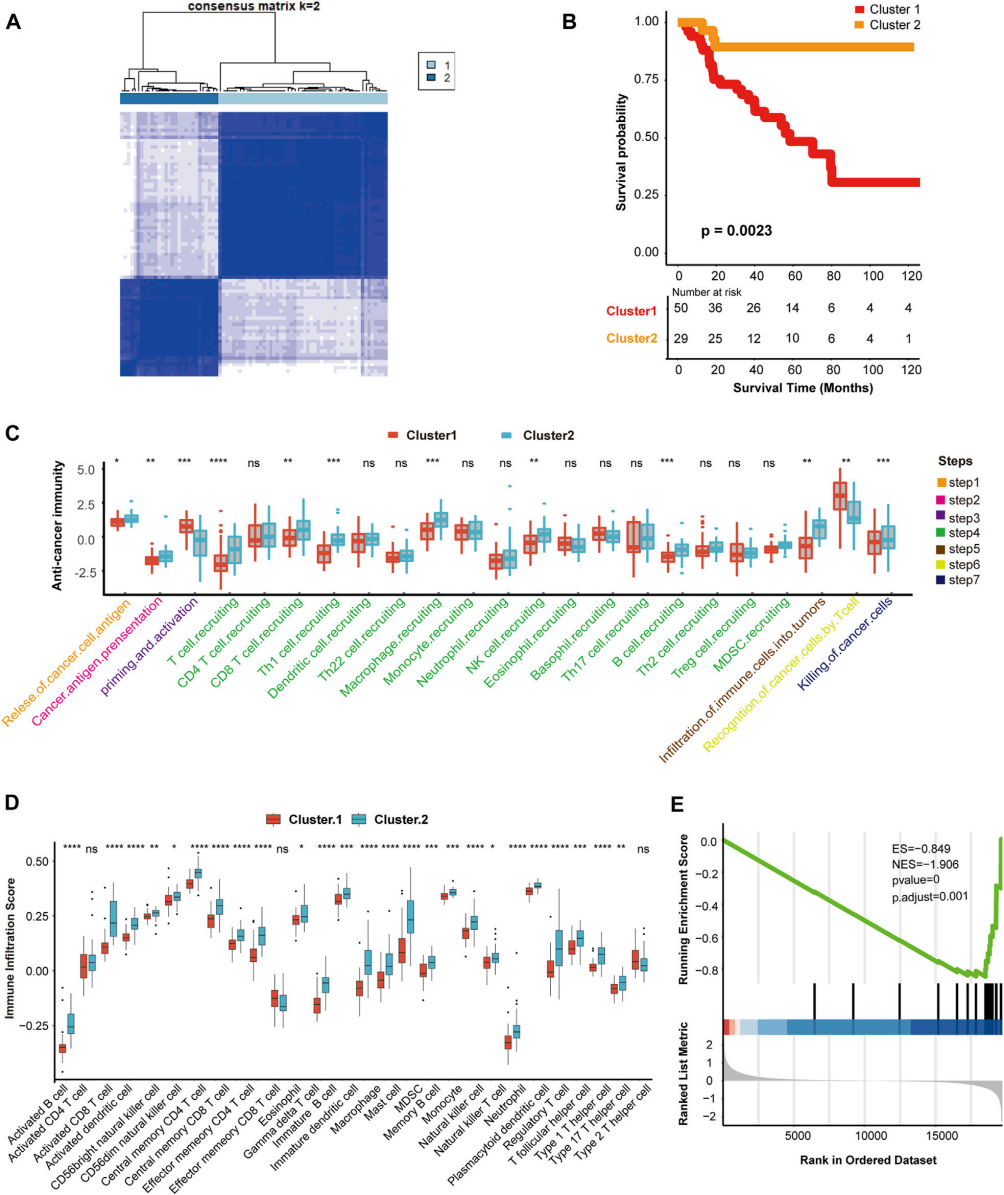
Hypoxia clusters correlated with immune phenotypes. **(A)** The TCGA-ACC cohort was divided into two distinct cluster when k = 2. **(B)** Survival analysis between cluster 1 and cluster 2. Cluster 1, red. Cluster 2, yellow. **(C)** Comparison of immune cycle activity involved in seven steps between cluster 1 and cluster 2. Cluster 1, red. Cluster 2, blue. **(D)** Comparison of various immune cell infiltration score between cluster 1 and cluster 2. Cluster 1, red. Cluster 2, blue. **(E)** The enrichment score of IFN-γ pathway in cluster 1 and cluster 2. |NES| > 1 and *p* < 0.01 were considered as statistically significant.

According to the expression of hypoxia-related genes, we found that when K = 2, unsupervised classification was the most effective. Hence, TCGA-ACC cohort was divided into two independent clusters: cluster 1 (50 patients) and cluster 2 (29 patients) (Figure 1A, Supplementary Figures S1 A–E). The survival analysis indicated that the overall survival (OS) rate in cluster 2 was greater than in cluster 1 (*p* = 0.0023) (Figure 1B).

Next, we explored the relationship between clustering and immune phenotypes. Throughout the immune cycle, several steps in cluster 2 involved releasing and presenting cancer cell antigens, extensive recruitment of immune cells, such as CD8 T cells, macrophages, B cells, natural killer (NK) cells, and killing of tumor cells, was more active in cluster 2 than cluster 1, so cluster 2 might be an inflammatory immunophenotype (Figure 1C). Furthermore, we compared the number of infiltrating immune cells between the two hypoxia clusters and found that the enrichment of NK cells, macrophages, dendritic cells (DCs), and neutrophils involved in the innate immune response in cluster 2 was significantly higher than cluster 1 (Figure 1D). In addition, activated CD8 T cells, B cells, or Th1 and Th17 cells connected with specific immunity were richer in cluster 2 than in cluster 1 (Figure 1D). Hypoxia cluster 2 was considered an inflammatory immune phenotype, whereas hypoxia cluster 1 was a non-inflammatory immune phenotype. In the anti-tumor immune cycle, IFN-γ is a pivotal modulator induced by active T cells and NK cells, and promotes cancer cell death (Alspach and Lussier, 2019). Our results showed that the IFN-γ pathway in cluster 1 was inhibited compared to that in cluster 2. Therefore, we reasonably presumed that cluster 2 was more sensitive to immunotherapy (Figure 1E).

### 3.2 Hypoxia related DEGs and functional analysis

**FIGURE 2:**
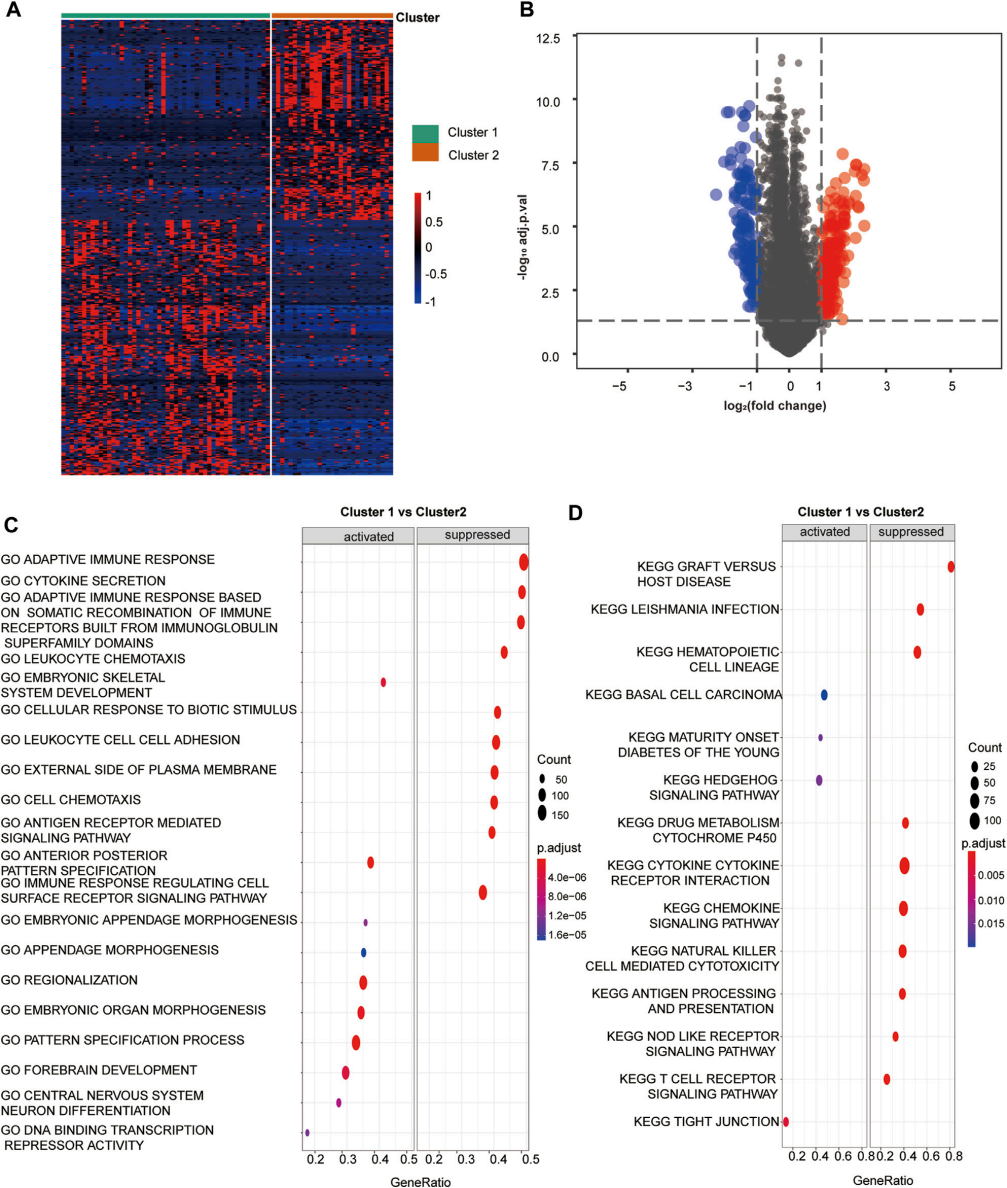
Hypoxia related DEGs and functional analysis. **(A)** A heatmap depicts the between DEGs cluster1 and cluster 2. Higher expression DEGs with are displayed in red, and lower expression are displayed in blue. **(B)** A volcano plot depicts the DEGs between cluster 1 and cluster 2. DEGs with log_2_(FC)≥1 were shown in red while the genes with log_2_ (FC)≤ -1 were shown in blue, and the genes with indiscriminate expression were shown in gray. **(C–F)** GO and KEGG analysis of DEGs between cluster 1 and 2. **(C)** The pathway in GO functional enrichment comparison between cluster 1 and cluster 2. **(D)** The pathway in KEGG functional enrichment comparison between cluster 1 and cluster 2.

We screened out 405 DEGs between the two hypoxia clusters, as shown in the heatmap (Figure 2A) and volcano plot (Figure 2B) (Supplementary Table S4). The expression levels of hypoxia-related genes between the clusters were almost the opposite. Furthermore, the GO analysis demonstrated the pathway containing adaptive immune response, leukocyte chemotaxis and adhesion, antigen receptor mediated signaling were suppressed in cluster 1 (Figure 2C). In addition, the T cell activation and regulation, T cell mediated cytotoxicity, signaling receptor and cytokine activity were down-regulated in cluster 1 (Supplementary Table S10). According to the results of the KEGG analysis, pathways involving chemokine signaling, natural killer cell mediated cytotoxicity, antigen processing and presentation and T cell receptor signaling were suppressed in cluster 1 (Figure 2D) (Supplementary Table S11). In view of the above results, we provided favorable evidence that hypoxia-related DEGs between the two clusters are strongly linked to immune activity and the immune response pathways were restrained in cluster 1 compared to cluster 2, which further confirmed that cluster 2 was more immunologically active.

### 3.3 Development of the HRS and its role in clinical prognosis prediction

**FIGURE 3:**
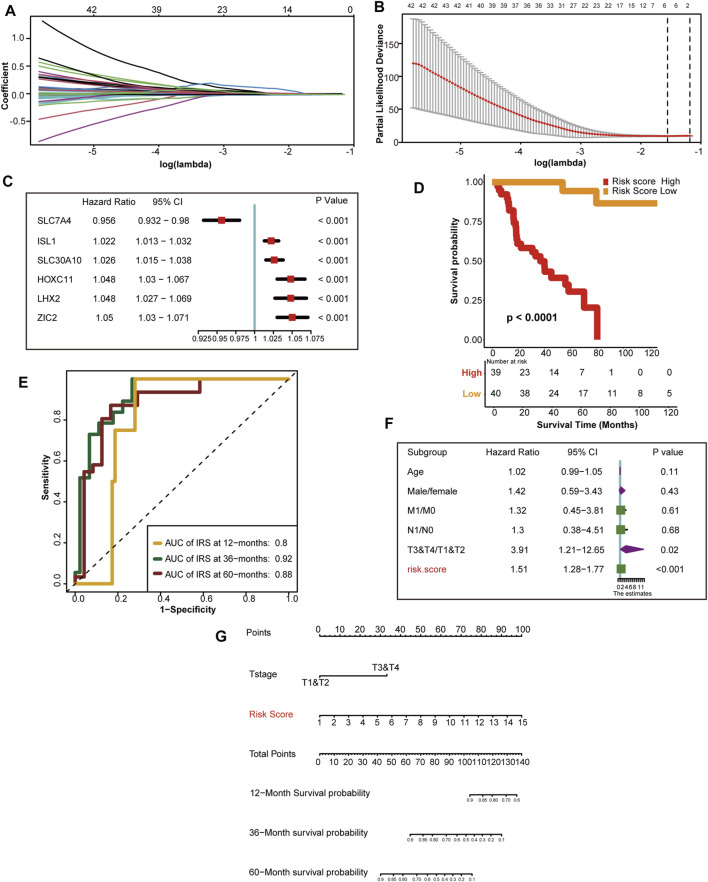
Development of HRS and the role in clinical prognosis prediction. **(A)** LASSO coefficient profiles of 143 hypoxia-related prognostic DEGs **(B)** Ten-fold cross-validation for tuning parameter selection in the LASSO model. The two dotted vertical lines are drawn at the optimal value using the minimum criteria. Optimal hypoxia genes with the best discriminative capability (6 in number) were selected for generating the HRS **(C)** Forest plot of hazard rations for six optimal hypoxia-related prognostic genes. **(D)** Survival analysis between the two different risk score group. Risk score high is shown red and risk score low group is shown yellow. **(E)** The predictive accuracy of the HRS for survival. **(F)** Results of univariate Cox analysis by integrating the HRS and clinicopathological characters. **(G)** The nomogram used to predict the 12-month,36-month, 60-month overall survival.

First, we selected 143 hypoxia related genes that were independently associated with the prognosis of TCGA-ACC discovery cohorts (Supplementary Table S7). Next, we screened out the optimal predictive genes: SLC7A4, ISL1, SLC30A10, HOXC11, LHX2, and ZIC2 (Figures 3A, B). Figure 3C shows that these six signatures were independent prognostic factors, and all but SLC7A4 were risk factors. Ultimately, we generated an HRS and used it to divide TCGA discovery cohort into high and low HRS groups. Obviously, patients in the low-risk group had better survival than those in the high-risk group (Figure 3D). In addition, the accuracy of the HRS in predicting 1-, 3-, and 5-year OS was 0.8 0.92, and 0.88, respectively (Figure 3E). Based on the excellent predictive performance, we combined the HRS with clinical characteristics for predicting the clinical outcome. A multivariate Cox analysis revealed that tumor stage and HRS were crucial independent risk factors (Figure 3F). Furthermore, our nomogram showed that the later the clinical stage and the higher the HRS, the worse the prognosis (Figure 3G). To be more convincing, we also tested whether the input data of independent prognostic analysis follow PH assumption. All of them follow PH assumption as expected (Supplementary Figure S10A) (Supplementary Table S12). Besides, we have analyzed the relationship between HRS and the clinicopathological characteristics of the patients. HRS were found to be significantly higher in high T and M stages, which indicates the important clinical predictive value. It also suggests that hypoxia in tumor microenvironment is more serious in ACC with higher stage (Supplementary Figure S2). Moreover, the DCA of nomogram indicated that the nomogram model we constructed was feasible to make valuable and profitable judgments for the survival rate of 3 or 5 years (Supplementary Figure S10B).

### 3.4 External validation of the HRS

**FIGURE 4:**
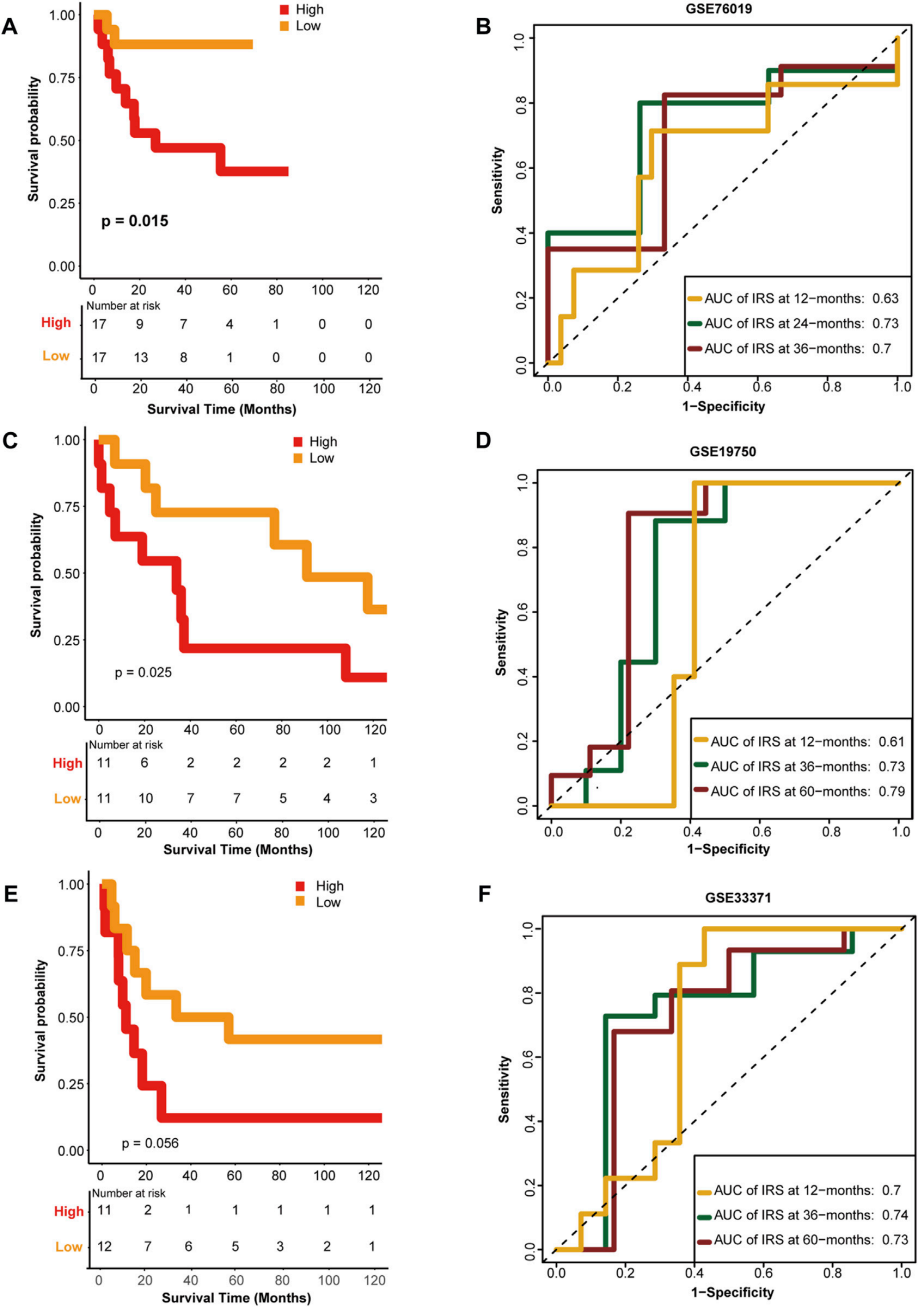
External validation of the hypoxia risk score. **(A, B)** Validation of the hypoxia risk score in GSE76019 **(C, D)** Validation of the hypoxia risk score in GSE19750 **(E, F)** Validation of the hypoxia risk score in GSE 33371.

To confirm the performance of the HRS, the GSE76019, GSE19750, and GSE33371 cohorts were used for external validation. Unsurprisingly, compared with the low-risk group, patients in the high-risk group had worse OS in the GSE76019 cohort (Figure 4A). The predictive accuracies for 1-, 3-, and 5-year OS were 0.63, 0.73, and 0.7, respectively (Figure 4B). In GSE19750, the OS of the high-risk group was poorer than that of the low-risk group (Figure 4C), and the predictive accuracies for 1-, 3-, and 5-year OS were 0.61, 0.73, and 0.79, respectively (Figure 4D). Consistently, the high-risk score group showed a lower OS rate in GSE33371 (Figure 4E). The predictive accuracies for 1-, 3-, and 5-year OS were 0.7, 0.74, and 0.73, respectively (Figure 4F). Besides, we have made multivariate regression analysis in the GEO datasets. Unfortunately, we only verified the independent prognostic value of HRS in GSE76019, but not in the other two GEO datasets (Supplementary Figure S3).

### 3.5 HRS correlated with the immune phenotype and immune checkpoint molecule expression

**FIGURE 5:**
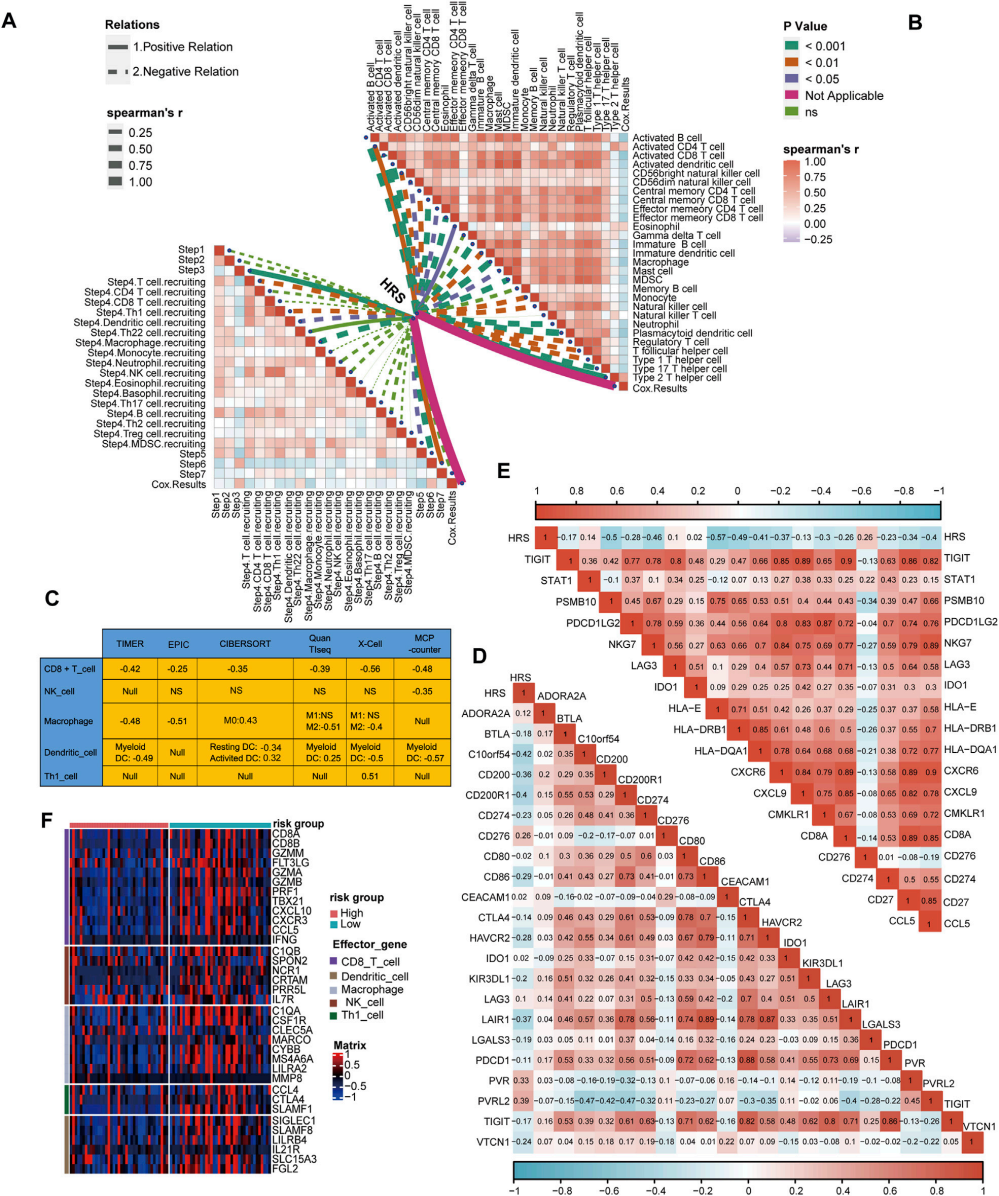
Differences in immunological characteristic between HRS groups. **(A, B)** Spearman correlation analysis of HRS with activity of cancer immunity cycle and immune cell in TME analyzed by ssGSEA. The positive correlation is shown in solid line. The negative correlation is shown in dotted line. The association strength was represented by the thickness of the lines. The different colors of the lines represent different *p*-values. **(C)** The associations between the HRS and the several anti-tumor immune cell in six different algorithms. **(D)** The correlations between HRS and immune checkpoint. **(E)** The correlations between HRS and T cell inflamed score. **(F)** A heatmap was drawn to depict the differences in cytotoxic effector molecule between high-risk score group and low-risk group.

Similarly, we connected the HRS to immune cycle activation. As expected, several steps involved in the immune cycle, including T cell recruitment, Th1 cell recruitment, DC recruitment, macrophage recruitment, and infiltration of immune cells, were negatively correlated with the HRS (All *p* < 0.05) (Figure 5A) (Supplementary Table S6). In addition, the infiltration of most antitumor immune cells, including activated B cells, activated CD8 T cells, activated DCs, memory T cells, macrophages, and NK cells, were inversely related to the HRS (All *p* < 0.05) (Figure 5B) (Supplementary Table S6). To be persuasive, an additional six algorithms were used (Supplementary Figures S4–S9). Overall, all algorithms indicated that CD8 T cells were lacking in the high HRS group. In addition, many of the algorithms showed macrophages and DCs were negatively correlated with HRS (All *p* < 0.05) (Figure 5C). Based on these findings, we infer that the low HRS group exhibited an inflammatory phenotype with prominent anti-tumor ability. Inflammatory phenotype tumors generally show high expression of immune checkpoint molecules (Trujillo et al., 2018). Indeed, the HRS was negatively correlated with the expression of several common immune checkpoint molecules such as CD274 (PD-L1), CD200, CTLA-4, TIGIT (All *p* < 0.001) (Figure 5D) (Supplementary Table S8). The gathering factors involved in the T cell inflamed score also suggest an inflammatory TME (Hu et al., 2021). Consistently, HRS was negatively related to T cell inflammatory score (TIS) (All *p* < 0.01) (Figure 5E) (Supplementary Table S8). Effector molecules of cytotoxic lymphocytes play an indispensable role in killing tumor cells (Martinez-Lostao et al., 2015). Thus, we found that most of the effector molecules were significantly increased in the low HRS group (Figure 5F). It was regrettable that HRS was positively correlated with TMB and not related to MSI (Supplementary Figures S10C, D).

## 4 Discussion

Hypoxia is a pivotal hallmark in most solid tumors owing to an imbalance in oxygen supply and demand (Jing et al., 2019). Throughout the process of tumorigenesis, hypoxia plays an indispensable role in cell-intrinsic oncogenic and TME suppression, which induces changes in cellular biological functions, ultimately leading to poor prognosis (Roma-Rodrigues et al., 2019). On the one hand, hypoxia contributes to cancer cell phenotype changes such as the epithelial-to-mesenchymal transition, resulting in invasion and metastasis (Hapke, 2020). On the other hand, HIF-α overexpression not only drives the activation of immune-suppressor cells, such as T regulatory cells, but also inhibits anti-immune cell and antigen-presenting actions involving CD8 T cells, NK cells, and DCs. This tends to impair immune surveillance and anti-tumor ability subject to the TME (Vaupel and Multhoff, 2018). In this study, we constructed a hypoxia risk assessment that might serve as an overall immune status predictor in ACC for evaluating immune phenotype and the response to immune checkpoint inhibitors (ICIs) to ultimately achieve ACC patient prognosis prediction.

Due to the development of bioinformatics, numerous studies have uncovered the effect of hypoxia gene signatures and pathways in the progression, prognosis, and curative effects in various tumors. Zhang et al. selected three hypoxia-associated genes (PDSS1, CDCA8, and SLC7A11) to construct a model for liver cancer diagnosis, prognosis, and recurrence (Zhang et al., 2020a). In advanced and high-risk clear cell renal cancer, Chen et al. found that the expression of four hypoxia-related long non-coding RNAs was decreased (Chen et al., 2021). Liu et al. discovered that HIF1α upregulation in glioma was associated with disease severity and drug resistance (Liu et al., 2020a). In triple-negative breast cancer patients, Yang et al. established a comprehensive index of hypoxia and immune genes for risk stratification (Yang et al., 2021b). In our study, we selected six optimal hypoxia-related genes. Among these, ISL-1 was reported to drive gastric and breast cancer progression (Zhang et al., 2018; Guo et al., 2019). The long non-coding RNA-SLC30A10 was associated with the colorectal tumor proliferation (Hou et al., 2020). Cui et al. (2020) found that HOXC11 functions as a novel oncogene in colon adenocarcinoma and kidney renal clear cell carcinoma. The down-regulating of LHX2 was able to inhibit the nasopharyngeal carcinoma growth (Liang et al., 2019). Besides, the role of ZIC in different tumors remains controversial (Lu et al., 2017; Liu et al., 2020b). Nevertheless, the relationship between these predictive genes and ACC had not been reported.

The morbidity of adrenal neoplasms is about 3%–10% (Else et al., 2014), but only a minority are malignant (Crona, 2019). Furthermore, the 5-year survival for ACC *in situ* is approximately 60%–80%, though this is reduced sharply to 30% in advanced ACC, and the recurrence rate is up to 75% even after complete resection (Fassnacht et al., 2009; Fassnacht et al., 2010). Recently, increasingly studies have focused on targeted therapy for ACC. Ruggiero et al. revealed that hampering VAV2 may be a new approach to inhibit metastatic progression in ACC (Ruggiero et al., 2017). Fiorentini et al. found a 17α-hydroxylase inhibitor capable of restraining the vitality of ACC (Fiorentini et al., 2016). Nevertheless, the curative effect of ICIs in patients with ACC remains controversial. Parise et al. found that high CD8T cell counts in pediatric ACC are persuasive evidence for immune response activation (Parise et al., 2019). In a phase 1b clinical trial, Le Tourneau et al. (2018) discovered that some patients could benefit from PD-1/PD-L1 inhibitors. In contrast, Fay et al. (2015) reported that PD-L1 expression in ACC was not associated with clinical pathology parameters or the survival rate. At the same time, some studies have found that patients with ACC have an inconsistent prognosis in response to immunotherapy related to immune escape, molecular alterations and the level of glucocorticoid (Araujo-Castro et al., 2021). Consequently, it is essential to identify patients with ACC who are responsive to immunotherapy.

In our study, we clustered ACC patients with 191 hypoxia-related genes into immune inflammatory and non-inflammatory phenotypes, indicating that hypoxia is a potential prognostic factor for ACC. However, the number of genes that needed to be tested was too large to be feasible. As a result, we selected six optimal gene signatures for constructing the risk score by Random Forests, though there are also many manuscripts recommending the LASSO method (Tibshirani, 1997; Zhang et al., 2020b). Random Forests harbors the property of collecting vital predictors without restricting their pairwise correlation, while the mutual exclusion of highly correlated variables in LASSO modeling is likely to skip crucial variables that cause predictive performance impairment (Zhu et al., 2015). We found that the HRS can also be used to effectively evaluate the prognosis of patients with ACC. In addition, the tumor stage is generally negatively correlated with prognosis. According to the results, we discovered that both tumor stage and HRS were independent risk factors for ACC. Therefore, we can infer that patient with advanced stage ACC and a high HRS are expected to have a worse prognosis.

Subsequently, we evaluated whether the HRS predicted performance in the immune status and ICI response. Our results showed that the HRS was negatively associated with anti-cancer immune activity and immune cell infiltration. However, it is worth noting that M2 macrophages have a negative correlation with HRS in our study. A large number of studies have shown that M2 macrophages play an important role in the microenvironment of tumor immunosuppression (Xia et al., 2020). Nevertheless, the focus of our study is on the inflammatory and non-inflammatory typing of the tumor microenvironment. But the increase of anti-inflammatory immune cells, such as M2 macrophages, making no influence on overall inflammatory microenvironment. Hence, we infer that there is more infiltration of various pro-inflammatory immune cells in the inflammatory tumor microenvironment, resulting in the corresponding increase of anti-inflammatory immune cells. Furthermore, the majority of factors involved in TIS were low in high HRS status, among which CCL5 and CXCL9 strongly enhanced CD8 T cell chemotaxis (Romero et al., 2020). Collectively, the high HRS group indicated that the non-immune phenotype presented with increased anti-tumor immunity, while the low HRS group showed a reverse trend. Increasing evidence suggests that ICI treatment is more effective in patients with an inflammatory phenotype (Nishino et al., 2017; Petitprez et al., 2020). In patients with ACC, we found no significant correlation between PD-1 and HRS, probably because of inadequate clinical data. Nevertheless, it should be point out that the expression of several other critical immune checkpoint molecules, such as PD-L1, CD200, CTLA-4 and TIGIT were negatively correlated with HRS. Hence, we believe that ICIs are a potential and promising treatment for ACC patients with low HRS. However, the prediction ability of HRS in TMB and MSI were limited on account of inconsistent inspection standards and different algorithms. Combined with other results illustrated in our texts, HRS was able to predict the TMB subtypes effectively. Regrettably, since there is no data on ACC immunotherapy, we were not able to directly use HRS to predict the efficacy of immunotherapy, which is one of the limitations of our study.

It should be pointed out that there were several other limitations in our study. Primarily, all of our data came from public databases. Thus, the experimental evidence and clinical material were not directly available. Second, the credibility of our findings is weakened because of the rarity of ACC medical records. Third, we set the median as the optimal cutoff value of the HRS. This was a subjective decision and requires further careful consideration.

## 5 Conclusion

In summary, our study revealed that hypoxia is associated with immune status in ACC. Taking this into account, we developed an HRS for use of risk classification to predict the immune phenotype, ICI response, and prognosis in ACC. It not only provides novel prognostic indicators for ACC but also offers some promising treatment targets for this disease.

## Data Availability

The original contributions presented in the study are included in the article/Supplementary Material; further inquiries can be directed to the corresponding authors.

## Glossary

TME: tumor immune microenvironment
ACC: Adrenocortical carcinoma
TIS: T cell-inflamed score
ICI: Immune checkpoint inhibitor
HRS: hypoxia risk score
ROC: receiver operating feature
AUC: area under the ROC curve
RSF: Random survival forest
GSEA: Gene Set Enrichment Analysis
FPKM: fragments per kilobase per million mapped fragments
TPM: transcripts per kilobase million
FDR: false discovery rate
log2 FC: log2 Fold change
KECG: Genes and Genomes
GO: Gene Ontology
BP: biological process
CC: cellular component
MF: molecular
GEO: Gene Expression Omnibus
MSigDB: Molecular Signatures Database
ssGSEA: single-sample gene-set enrichment analysis interferon-γ (IFN-γ)
LASSO: least absolute shrinkage and selector operation
K-M: Kaplan-Meier
DEGs: differentially expressed genes
OS: Overall survival
TNM: Tumor Node Metastasis
Th1 cell: Type 1 T helper cell
Th17 cell: Type 17 T helper cell NK: nature killer
DC: dendritic cell
CTL: cytotoxic T lymphocyte.
MSI: Microsatellite instability
TMB: tumor mutational burden
DCA: decision curve analysis
HIF-1α: hypoxia-inducible factor 1α
PD-1: programmed death-1
PD-L1: programmed death-ligand 1
M2: Macrophage 2.
