# Supplementary figures and images for: A hypoxia risk score for prognosis prediction and tumor microenvironment in adrenocortical carcinoma
Source: Front Genet. 2022 Dec 13;13:796681. doi: 10.3389/fgene.2022.796681 (PMC9792869; doi:10.3389/fgene.2022.796681)

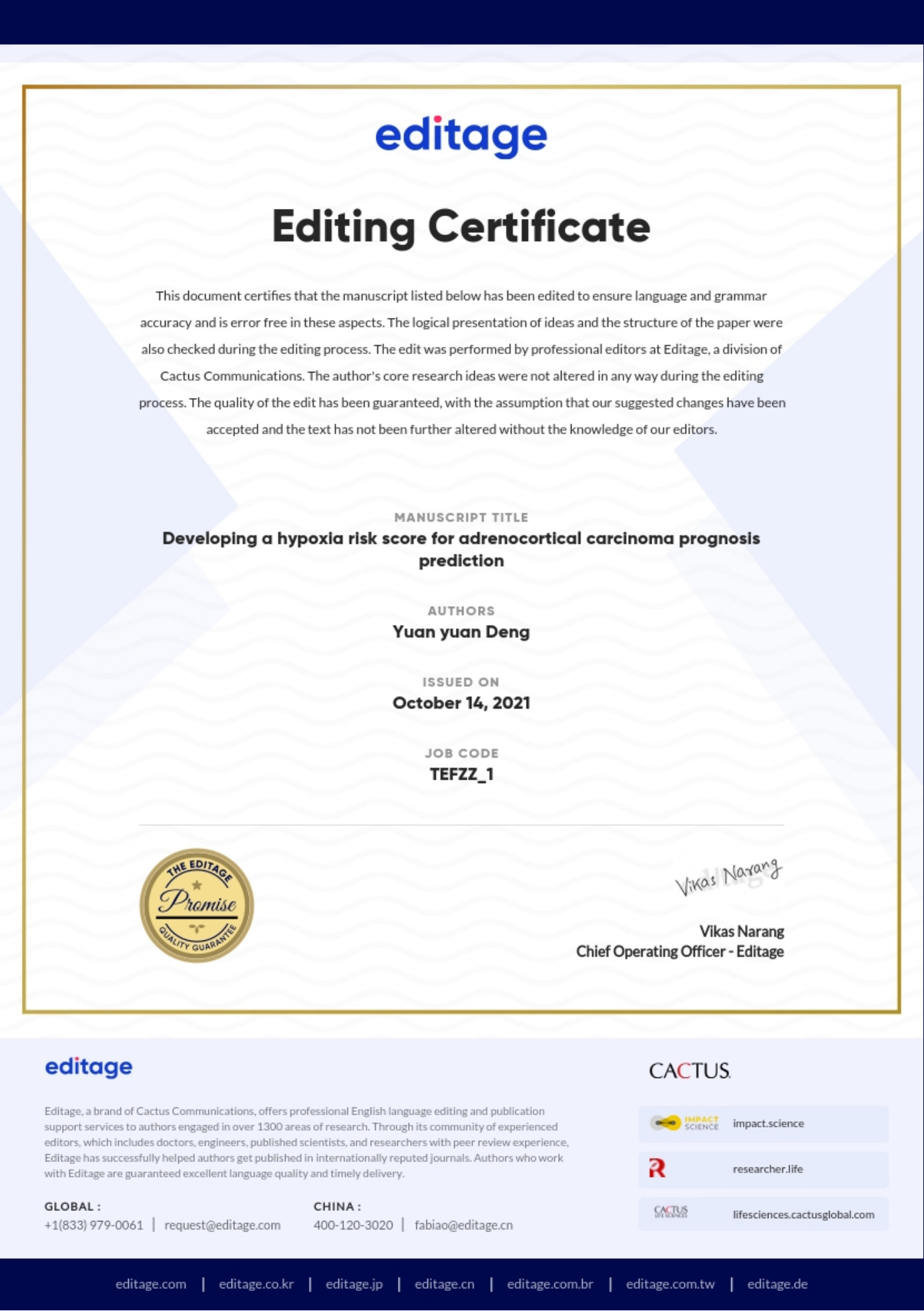

Supplement: Supplementary file 4 [file Image11.TIF]

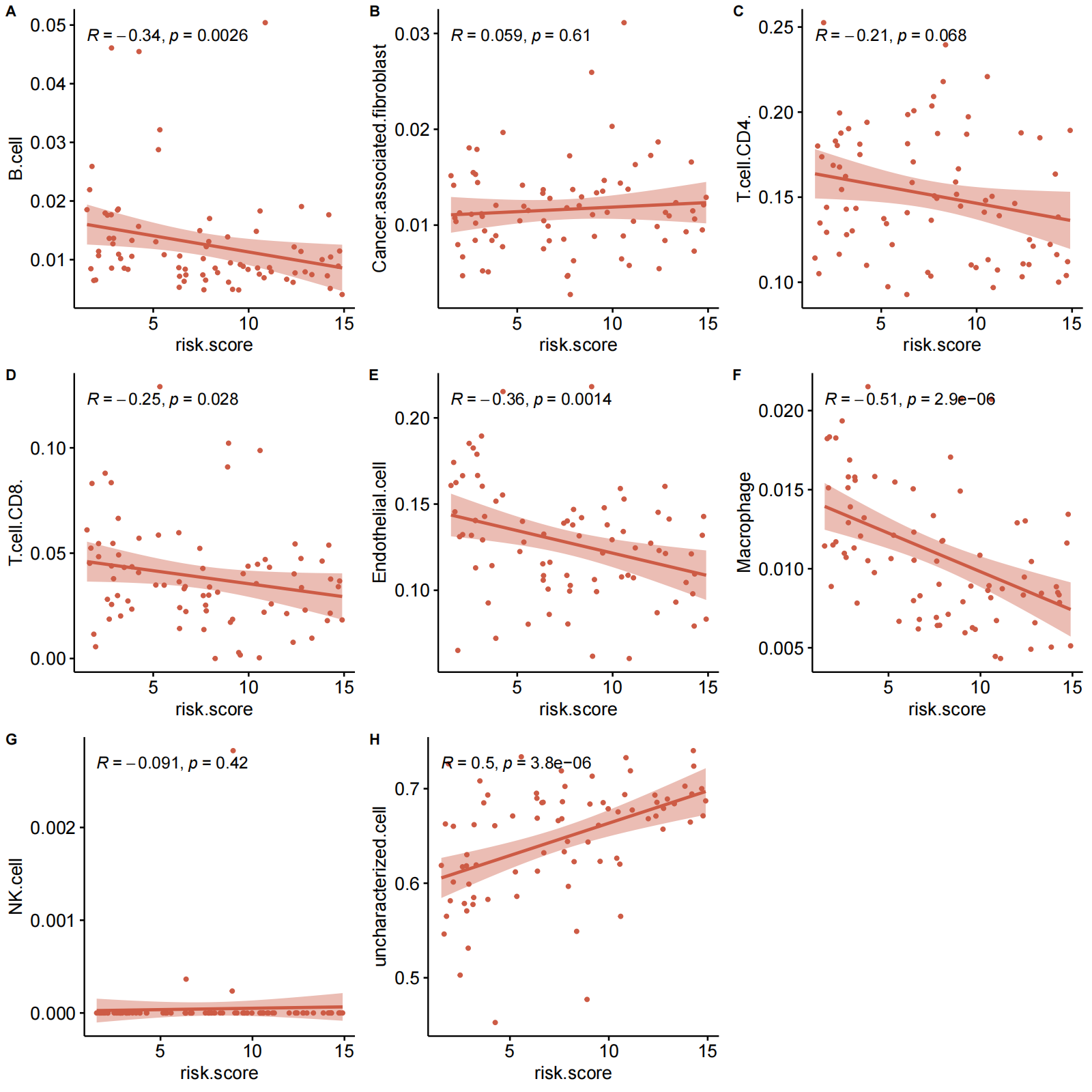

Supplement: Supplementary file 5 [file Image5.PNG]

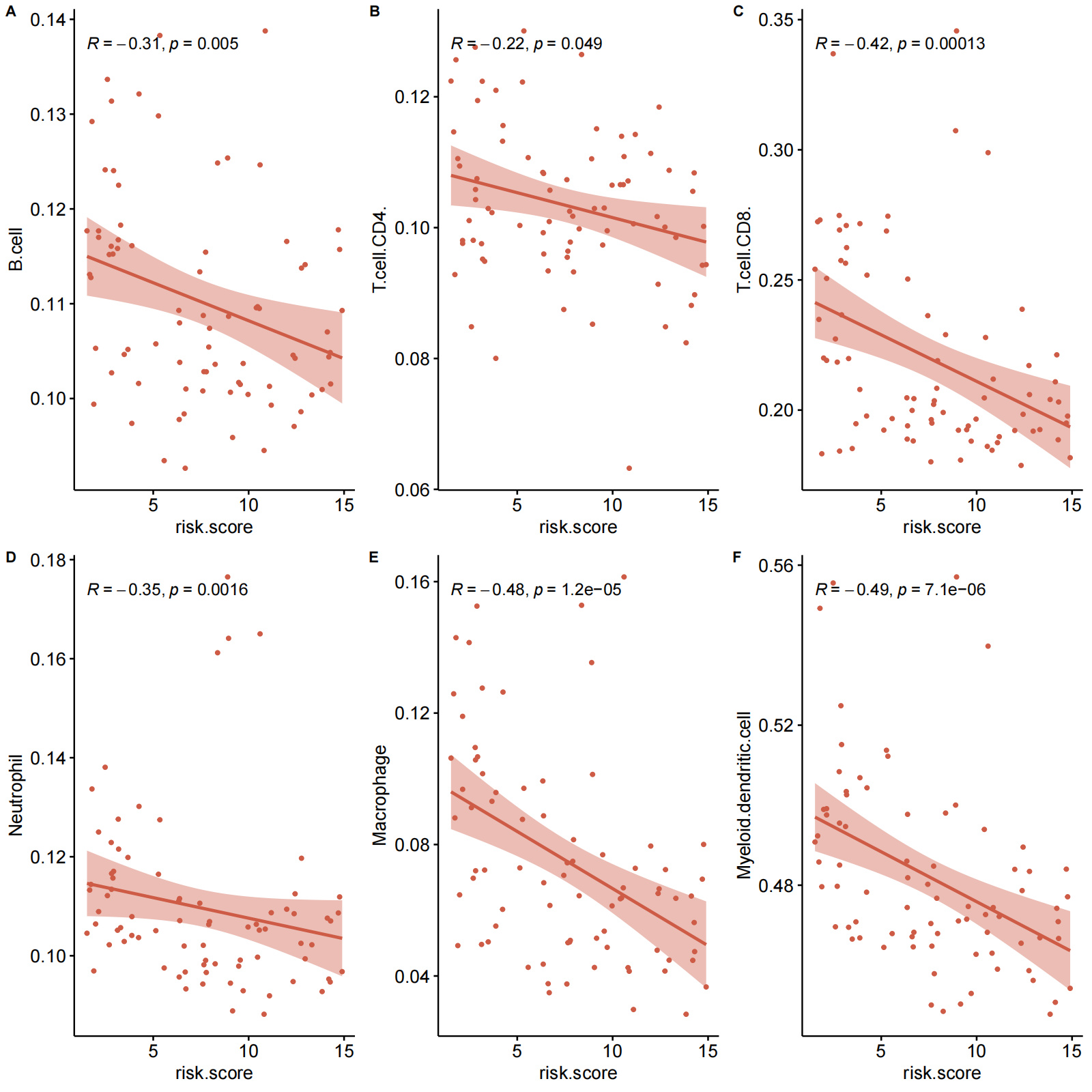

Supplement: Supplementary file 6 [file Image4.PNG]

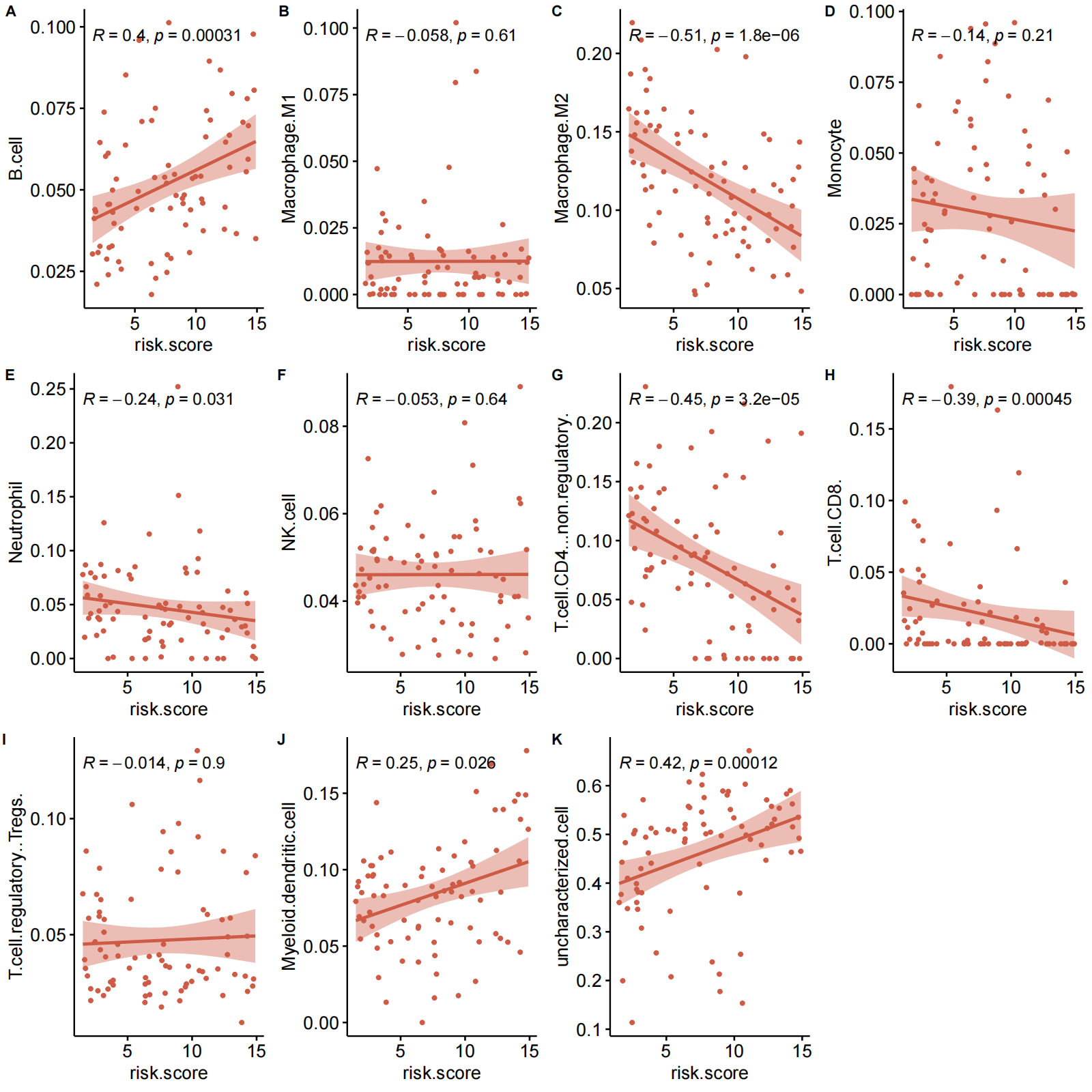

Supplement: Supplementary file 10 [file Image7.PNG]

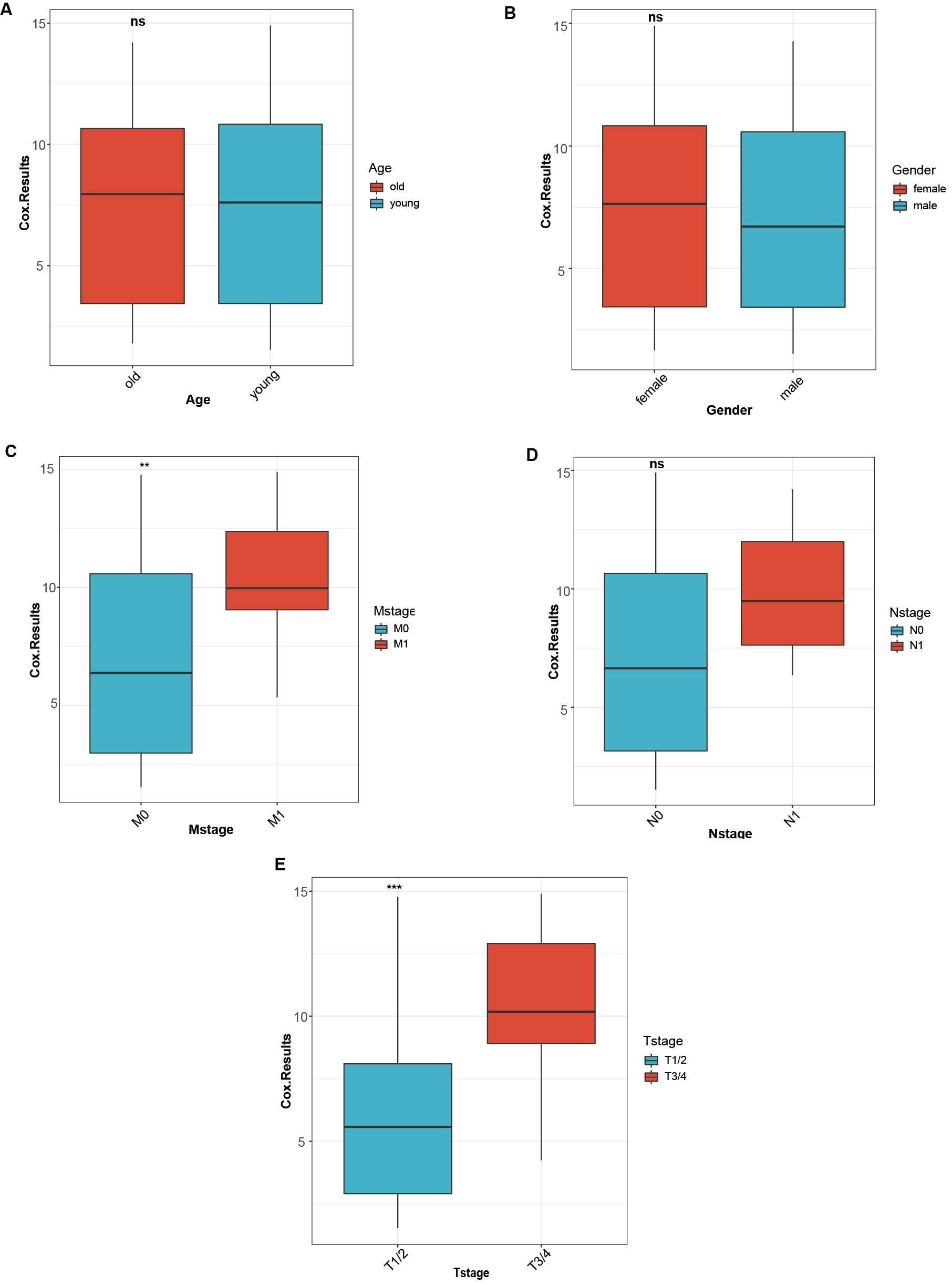

Supplement: Supplementary file 11 [file Image2.PNG]

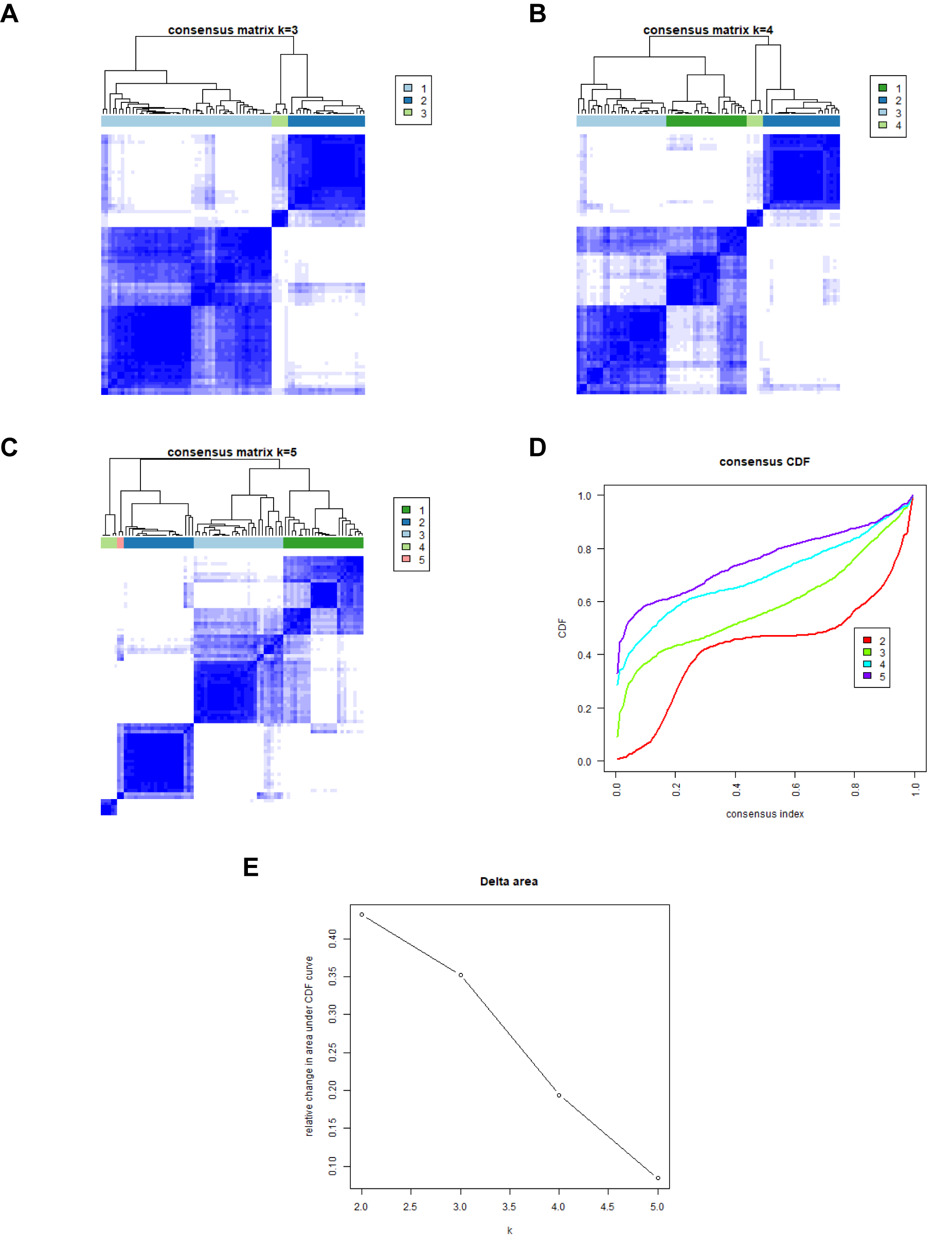

Supplement: Supplementary file 13 [file Image1.PNG]

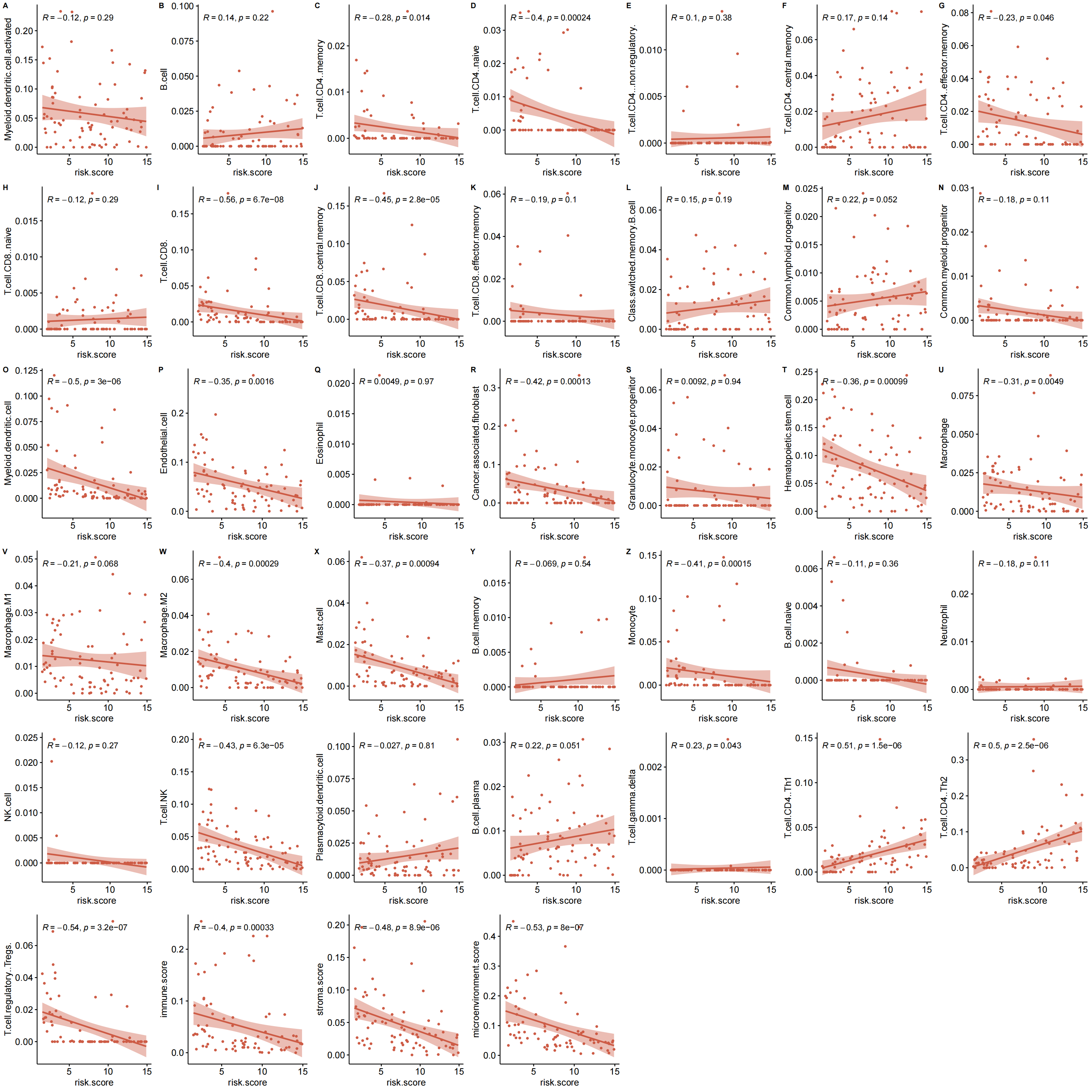

Supplement: Supplementary file 14 [file Image8.PNG]

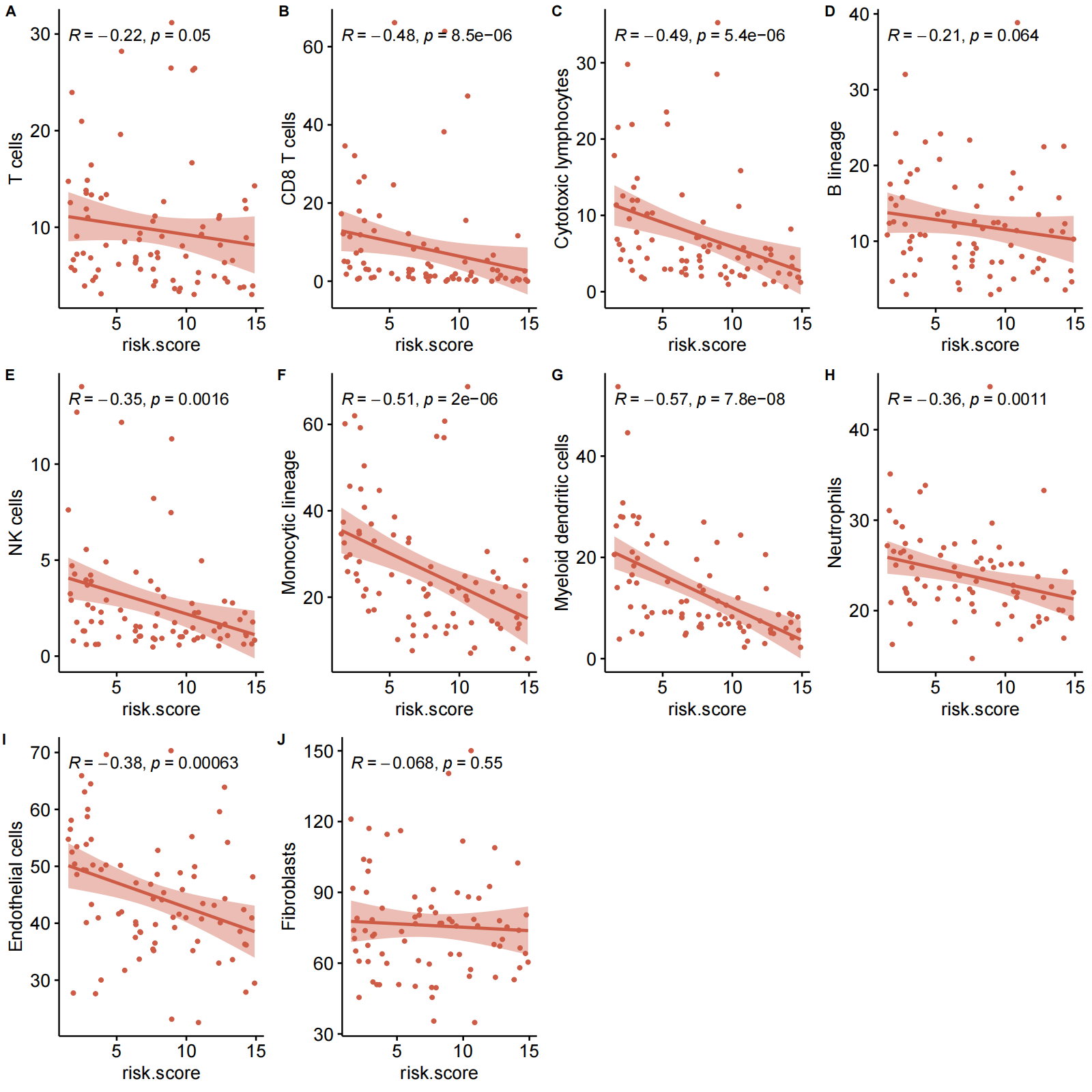

Supplement: Supplementary file 15 [file Image9.PNG]

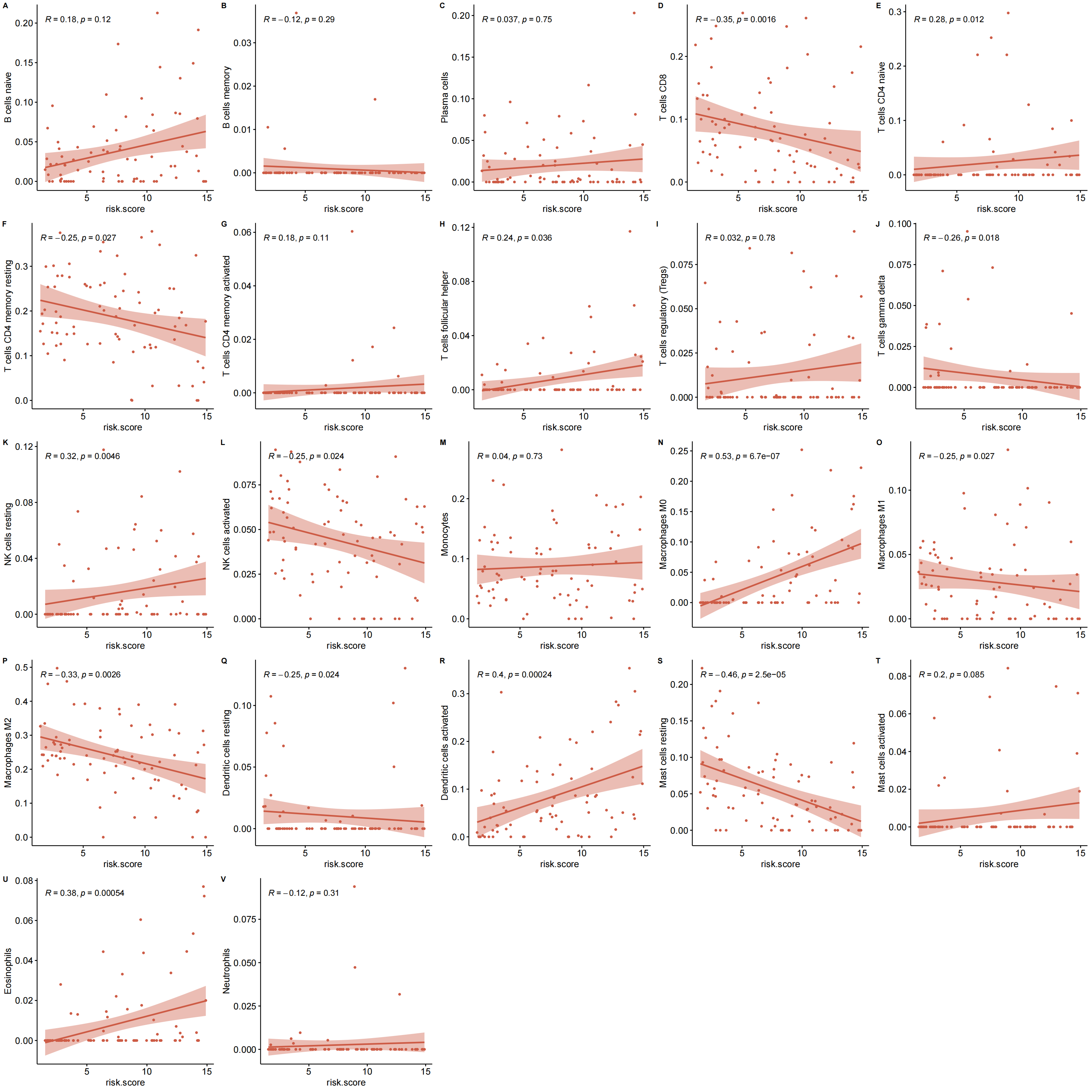

Supplement: Supplementary file 19 [file Image6.PNG]

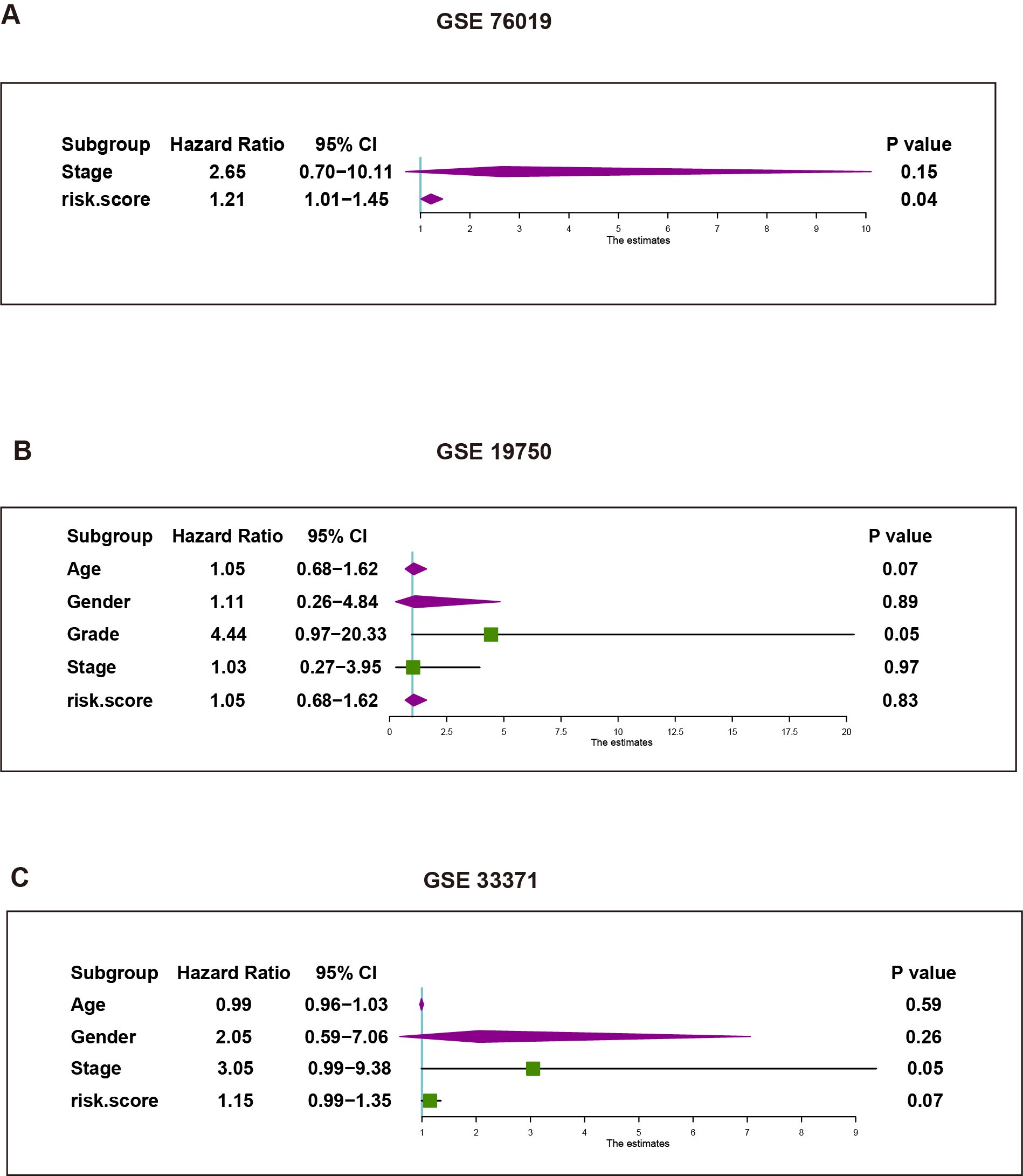

Supplement: Supplementary file 22 [file Image3.PNG]

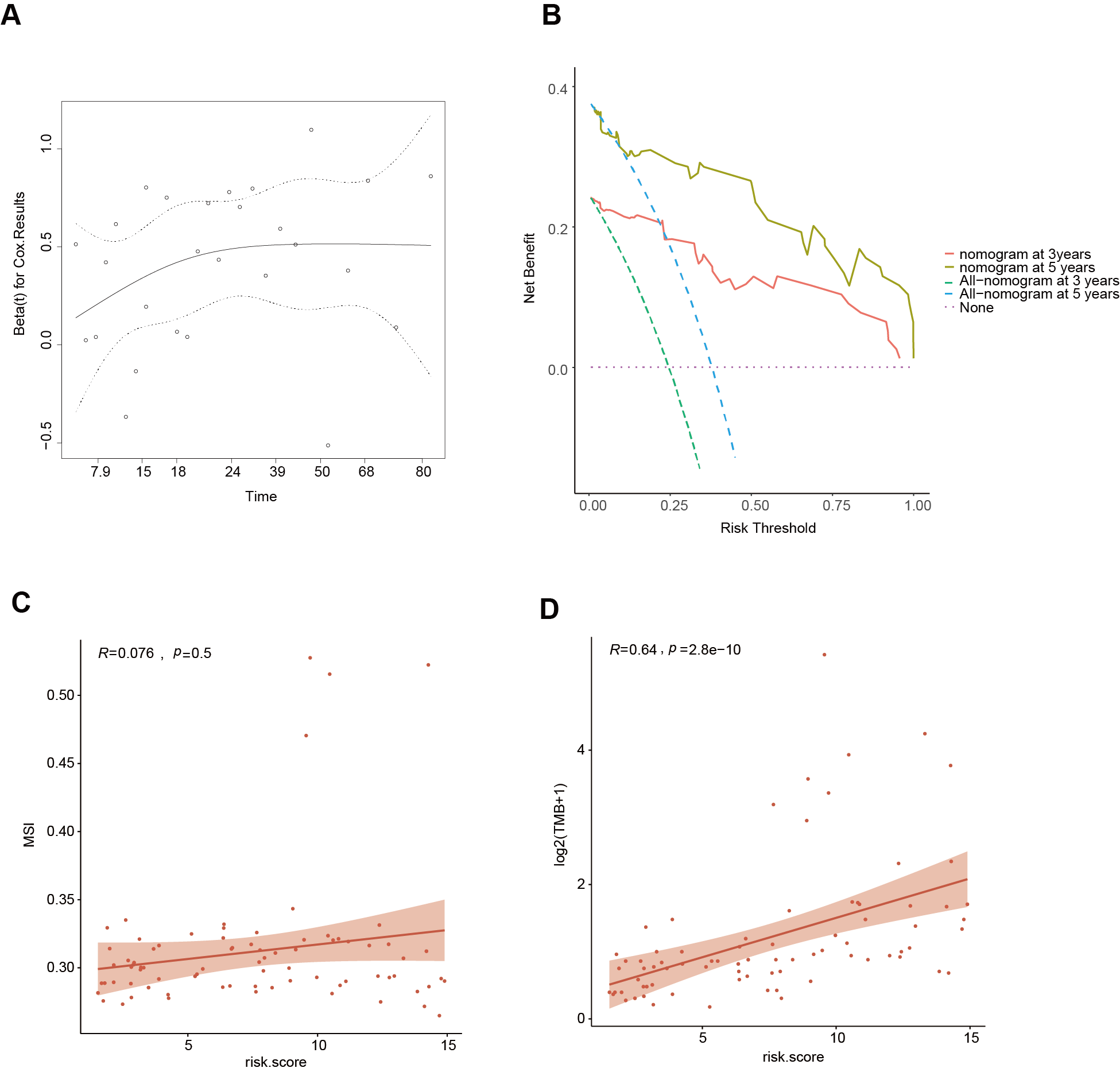

Supplement: Supplementary file 23 [file Image10.PNG]
